# Cross-linked polyvinyl alcohol-xanthan gum hydrogel fabricated by freeze/thaw technique for potential application in soft tissue engineering

**DOI:** 10.1039/d2ra02295h

**Published:** 2022-08-05

**Authors:** Sergio Alberto Bernal-Chávez, Sergio Alcalá-Alcalá, Y. S. Tapia-Guerrero, Jonathan J. Magaña, María Luisa Del Prado-Audelo, Gerardo Leyva-Gómez

**Affiliations:** Departamento de Farmacia, Facultad de Química, Universidad Nacional Autónoma de México Ciudad de México 04510 Mexico q901108@gmail.com leyva@quimica.unam.mx; Laboratorio de Tecnología Farmacéutica, Facultad de Farmacia, Universidad Autónoma del Estado de Morelos Cuernavaca Morelos Mexico sergio.alcala@uaem.mx; Laboratorio de Medicina Genómica, Departamento de Genética (CENIAQ), Instituto Nacional de Rehabilitación-Luis Guillermo Ibarra Ibarra (INR-LGII) Ciudad de México 14389 Mexico yessicasarai@gmail.com jmagana@inr.gob.mx; Tecnologico de Monterrey, Escuela de Ingeniería y Ciencias, Campus Ciudad de México Mexico luisa.delprado@tec.mx

## Abstract

The search for materials and process parameters capable of generating hydrogels for soft tissue engineering applications, based on an experimental design strategy that allows the evaluation of several factors involved in their development and performance, has greatly increased. Nevertheless, the fabrication technique can influence their mechanical properties, swelling, crystallinity, and even their susceptibility to contamination by microorganisms, compromising their performance within the tissue or organ. This study aimed to evaluate the influence of the freeze/thaw technique on different characteristics of polyvinyl alcohol–xanthan gum hydrogel. Methods: this research analyzed the critical variables of the freeze/thaw process through a systematic study of a 2^*k*^ factorial design of experiments, such as the proportion and concentration of polymers, freezing time and temperature, and freeze/thaw cycles. Additionally, physicochemical analysis, susceptibility to bacterial growth, and cell viability tests were included to approximate its cytotoxicity. The optimized hydrogel consisted of polyvinyl alcohol and xanthan gum at a 95 : 5 ratio, polymer mixture concentration of 15%, and 12 h of freezing with three cycles of freeze/thaw. The hydrogel was crystalline, flexible, and resistant, with tensile strengths ranging from 9 to 87 kPa. The hydrogel was appropriate for developing scaffolds for soft tissue engineering such as the cardiac and skeletal muscle, dermis, thyroid, bladder, and spleen. Also, the hydrogel did not expose an *in vitro* cytotoxic effect, rendering it a candidate for biomedical applications.

## Abstract 2

A polyvinyl alcohol–xanthan gum hydrogel was developed and the influence of the freeze/thaw technique on different characteristics was evaluated.

## Graphical abstract

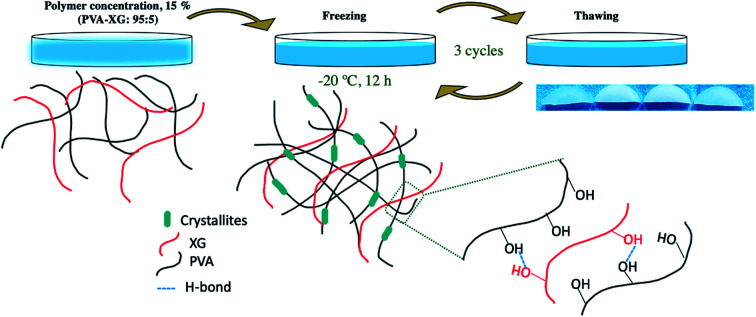

## 1 Introduction

By 2015, tissue engineering represented approximately 13% of regenerative medicine; however, it was estimated that by 2022 the market associated with tissue engineering will have grown by 300%.^1^ Tissue engineering substantially combines principles of alternative materials to replace damaged tissue or promote endogenous regeneration^2^ because the human body cannot regenerate large defects.^3^ Soft tissue involves skin, fat, muscle, fascia, tendon, cartilage, nerves, and vessels. In particular, soft tissues support and connect the functioning of the body structures and organs. Soft tissue damage is frequently related to diseases, trauma, and aging, while autologous treatment is not always available and is not constantly efficient. For this reason, soft tissue engineering is a strategy that seeks the reconstitution or regeneration of damaged or diseased tissue.^4^

Hydrogel-type polymeric matrices (HG) are biomaterials representing a high percentage of applications in tissue engineering (around 50% based on the review of Sepehr Talebian *et al.*^1^). In recent years, the technology has allowed the development of HG highly resistant, serving as platforms for the controlled release of active pharmaceutical ingredients, presenting characteristics such as low susceptibility to microbial contamination and high biocompatibility.^5–7^

Conventional HGs can present some defects that, over time, can increase, such as changes in pH, development of inflammatory events, or even becoming toxic to the underlying cells.^8^ Some properties frequently studied during the development of HG for tissue engineering are the surface chemistry, where the hydrophilicity/hydrophobicity balance allows the interaction with serum proteins by either promoting or inhibiting their adsorption and denaturation and mechanical behavior.^9^ Then, the control of the mechanical properties of these materials becomes a critical variable in the manufacturing of HGs, because it provides characteristics that mimic the mechanical properties of tissues, for instance, in the joints or tendons, which are tissues that are subjected to constant mechanical stress.^10,11^ The mechanical properties of HGs can be modified through the polymer concentration of the mixture and the cross-linking density from moduli below one kilopascal and up to moduli of several megapascals.^6^ The freeze/thaw (F/T) method used for developing HGs is a physical technique that avoids crosslinkers, potentially leading to the release of toxic agents.^12^ F/T consists of subjecting the polymer dispersion in the vehicle, generally aqueous, to freezing, usually below −15 °C, for a period that depends on the type of material and polymer concentration. Finally, the system is subjected to a thawing period, generally carried out at room temperature; this procedure becomes cyclical and is maintained as many times as necessary based on the objective sought.^13^ In the F/T process, water crystallization creates high polymer concentrations in interstitial domains. Polymer chains belonging to these highly concentrated domains could crystallize, leading eventually to the gelation of the whole system.^14^

Most HGs have been manufactured from natural polymers such as alginate, gelatin, chitosan, xanthan gum, hyaluronic acid, or synthetic polymers like polyethylene glycol and polyacrylic acids, polyvinyl alcohol, and polyacrylamides.^1,15,16^ However, natural polymers, related or similar to the extracellular matrix, generally present higher biocompatibility than synthetic ones, but synthetic materials present more resistance than natural polymers. Therefore, combining polymers is an attractive strategy to obtain biological advantages and, at the same time, obtain the flexibility and elasticity needed. In this context, xanthan gum (XG), a natural polymer, and polyvinyl alcohol (PVA), a synthetic polymer, have been used to develop HGs.^17–19^ However, PVA-based HGs are not flexible enough mechanically and often present fragility; therefore, PVA is often used in combination with other polymers to achieve the required characteristics of the gels.^20^ PVA contains multiple hydroxyl groups that can be easily induced to form a stable hydrogen bonding network; likewise, it has distinct characteristics: low cost, low toxicity, high mechanical strength, excellent biocompatibility, and chemical stability. While XG is rich in hydroxyl, carboxyl, and other functional groups, making it a polymer with the ability to interact with PVA, it is also biocompatible.^21^ Unfortunately, XG cannot provide a suitable platform due to its poor physical strength.^22^ Then, combining these two materials subjected to F/T cycles could generate a biomaterial with suitable mechanical properties, such as an increase in tensile strength (TS) that can be optimized to TS values appropriate for a target tissue varying the ratio of the polymers, the freezing temperature, the concentration of polymers and/or the number of cycles of F/T. The presence of hydroxyl groups in both polymers is a favorable characteristic since hydroxyl groups have presented high levels of cell adhesion.^23^

Moreover, it has been reported that negatively charged carboxyl groups, present in XG, can generate high reductions in inflammatory responses and are effective for fibroblast adhesion, inhibiting bacterial growth.^23,24^ The successful design of PVA-XG HGs can potentially extend the life and improve patient safety considering the physical, chemical, and toxicological characteristics that have been mentioned about these polymers. Therefore, understanding the mechanism by which these materials act is crucial for their design and potential use in tissue engineering. The design of experiments is a tool that can be used to evaluate the impact and the relationship between multiple input variables, obtain a greater understanding of the process, and develop a proper control strategy, as well as allow suitable resource management, promoting sustainable development and promotes advantages over evaluating one factor at a time.^25^ The development of hydrogels through the F/T technique depends on multiple factors that significantly influence the mechanical and chemical properties of the hydrogel.^26^

The PVA-XG HGs have been developed and subjected to F/T treatment;^21,27^ however, previous proposals have been oriented towards searching for PVA-XG HGs with mechanical properties for application in food packaging areas or wastewater treatment. Therefore, our development of the PVA-XG HG contemplates a systematic evaluation based on the optimization of the HG through a 2^*k*^ factorial experimental design that allows the assessment of several factors involved in the F/T technique for soft tissue engineering application. Those factors determine the properties of the biomaterial in a reliable interval in which the mechanical behavior can be guaranteed for optimal reproducibility. Finally, our HG proposal, in addition to the mechanical and chemical characterization, tries to provide evidence of the biomaterial's microbiological resistance and cell viability, which in areas of tissue engineering are essential and have not been reported. Based on the above, the focus of this project was to develop a hydrogel using PVA and XG by the F/T technique (PVA-XG), comparing it with an HG which was not subjected to the F/T technique (PVA+XG), as well as the evaluation and characterization of various properties of the formulation for the possible performance in soft tissue engineering were performed.

## 2 Results and discussion

### 2.1 Mechanical properties

**Fig. 1:**
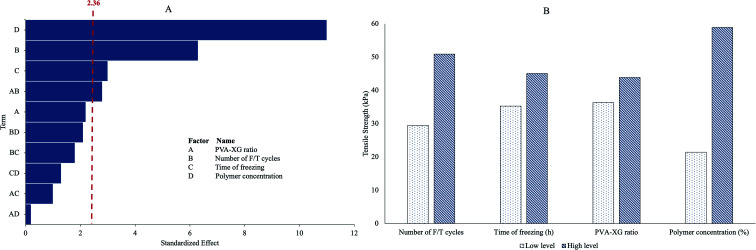
(A) Pareto chart of the standardized effects on the tensile strength (*α* = 0.05). (B) Average effect (mean ± SD, *n* = 8) on the tensile strength of the HGs associated with the change in the levels of each factor studied.

After executing the design matrix (19 runs, Table 1) and the statistical analysis, as shown in Fig. 1A, the variables polymer mixture concentration (*p* = 0.000), number of F/T cycles (*p* = 0.000), and time of freezing (*p* = 0.025) exhibited a significant effect on the TS of PVA-XG; meanwhile, the variable PVA-XG ratio only had a significant influence in interaction with the variable number of F/T cycles (*p* = 0.037).

**Table:** Design matrix for optimization of PVA-XG hydrogel and the measured tensile strengths with the physical appearance of each hydrogel

Run order	Factors (F)				Outcome Variable	
	PVA-XG ratio (% wt/wt)	Number of F/T cycles	Time of freezing (h)	Polymer concentration (%)	Tensile strength (kPa)	Physical appearance
1	85 : 15	3	3	10	21.656	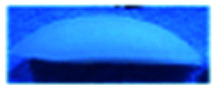
2	95 : 5	1	3	10	10.114	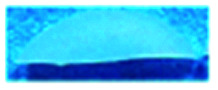
3	95 : 5	3	12	10	35.010	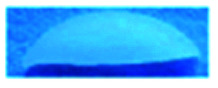
4	95 : 5	3	3	15	80.863	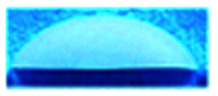
5	85 : 15	1	12	15	53.774	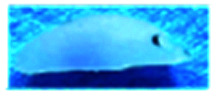
6	85 : 15	3	12	15	65.526	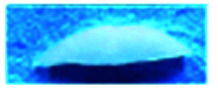
7	95 : 5	1	3	15	26.073	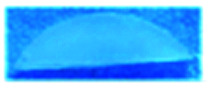
8	90 : 10	2	7.5	12.5	46.484	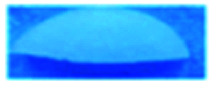
9	95 : 5	3	3	10	33.246	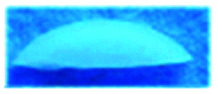
10	85 : 15	3	3	15	59.890	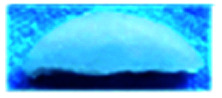
11	85 : 15	1	3	15	41.193	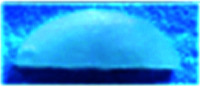
12	85 : 15	1	3	10	9.381	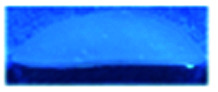
13	95 : 5	1	12	15	57.350	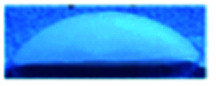
14	90 : 10	2	7.5	12.5	39.116	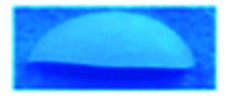
15	85 : 15	3	12	10	23.973	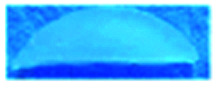
16	85 : 15	1	12	10	16.680	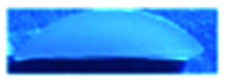
17	95 : 5	3	12	15	87.035	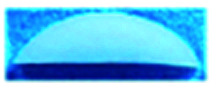
18	90 : 10	2	7.5	12.5	42.373	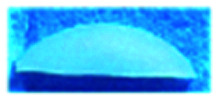
19	95 : 5	1	12	10	21.464	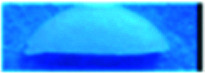

The TS, which varied from 9.038–87.03 kPa among the 19 runs, increases as the different factors studied increase. Concerning the PVA-XG ratio, an increase in the proportion of XG in PVA-XG decreases the TS. In contrast, an increment in the number of F/T cycles and proportion of PVA presented an increment in TS (Fig. 1B). The increase in TS is associated with augmented cross-linking due to crystallite formation, mainly by PVA, which allows the mechanical load distribution along the crystallites. The number and stability of these crystallites increase according to the number and time of F/T cycles increase.^17^ The crystallites act as physical cross-links to hold the network structure in the PVA-XG. If the PVA-XG breaks, the possibility of having broken crystallites on the cut surfaces cannot be ruled out.^17^

The optimization tool of Minitab® V.17 software found that 95 : 5 (PVA-XG) ratio, a polymer mixture concentration of 15% (14.25% for PVA and 0.75% for XG), and 12 h of freezing with three cycles of F/T generated material with appropriate flexible and resistant mechanical properties. The described properties were corroborated and compared with the estimated values from the statistical software, obtaining *p*-values greater than 0.05 (0.06328) for TS, confirming the equality between the observed and expected values (Table 2). According to the different TS values observed during experimentations, which were between 9 to 87 kPa, and, considering Young's modulus reported for various organs and tissues,^9^ it can be considered that the various HGs, including the optimized, can have an application to cardiac and skeletal muscle, dermis, thyroid, bladder, and spleen which have elastic modulus ranging from 7.5–50 kPa.^28^ Moreover, these organs' cells are soft entities with bulk elastic moduli in the 0.1–10.0 kPa range. In addition, after the neural tissues, the anatomical protection of the abdominal organs such as the bladder and spleen should be considered because they can be easily damaged. Therefore, the values obtained for TS are magnitudes that can contribute to their protection. It has been reported that, for example, spleen stiffness can increase from 15–20 kPa in healthy individuals to 50 kPa in patients with liver fibrosis;^28^ in this sense, the design space of the developed PVA-XG HG would cover such situations as well as other properties that are relevant for this type of biomaterials, such as porosity, surface chemistry,^23^ among others.

Optimization of the factors studied on the response variable tensile strength. **Mean ± CI, 95%, *n* = 3

**Table:** 

Factors (F)	Optimized level by Minitab® V.17
PVA-XG ratio (% wt/wt)	95 : 5
Polymer concentration (%)	15
Number of F/T cycles	3
Time of freezing (h)	12

**Table:** 

Outcome variable (OV)	Desirability	Estimated OV value	Observed OV interval**	*p*-value
Tensile strength	1.000	87.1 kPa	86.3 ± 0.2 kPa	0.0633
Composite desirability	1.000			

**Fig. 2:**
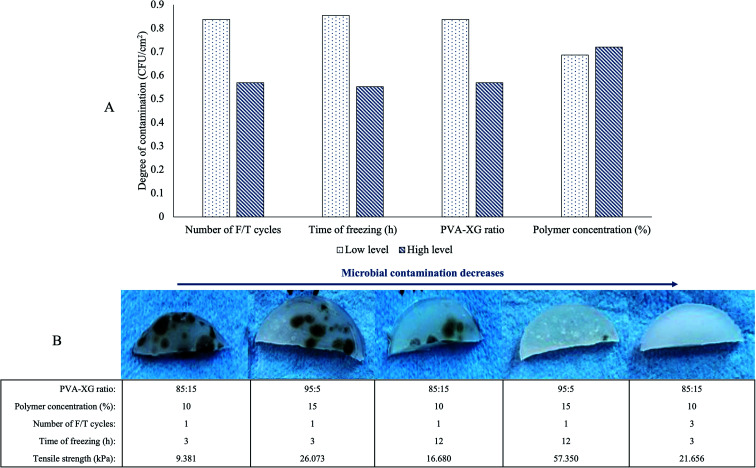
Susceptibility to contamination of PVA-XG hydrogels. (A) Degree of contamination in relation to the factors studied. The high and low levels represent the values at which each factor was tested in the experimental design. The bars show the average effect (mean ± SD, *n* = 8)) of each level for each factor. (B) The physical appearance of some HGs obtained during PVA-XG optimization under different experimental conditions and its susceptibility to microbial contamination after one month of being stored at 3 °C. The evidence corroborated in triplicate from each batch CFU: Colony Forming Unit.

An aspect observed during HG optimization is the susceptibility of some manufactured HGs to microbial contamination. In this sense, native XG poses more susceptibility to microbial contamination than PVA.^29^ Although all HGs manufactured were kept for 1 month at 3.0 ± 1.0 °C in a plastic dish after their F/T cycles and TS determination, some of the manufactured HGs presented contamination. Remarkably, by the physical appreciation of the hydrogel, it could be inferred that the factors studied also influence its conservation. Fig. 2B exhibits some HGs at room temperature after being stored for one month at 3 °C. We found a decrease in XG proportion (PVA-XG ratio from 85 : 15 to 95 : 5) and the increase in the number of F/T cycles (from 1 to 3 cycles) and freezing time (from 3 to 12 h) decrease the susceptibility to contamination of the hydrogel (Fig. 2A).

However, it is important to clarify that, even though the average effect of each factor (Fig. 2A) indicates how it influences HG contamination, the final effect is the result of a combination of the various levels of each factor studied and that are associated with each HG specifically. For example, the HG at the far right with an 85 : 15 ratio and a polymer concentration of 10% has less contamination compared to the first and third HG that show greater contamination with the same values; however, the number of cycles of F/T is different. For the optimized PVA-XG, no microbial contamination was observed after one month under the same storage conditions. This increase in the conservation of HGs may be related to the fact that a more significant number of F/T cycles, as well as a longer freezing time, increases the cross-linking, which decreases the capacity for the HGs to take up water, causing a decrease in the water activity and thus, less susceptibility to contamination. However, a limitation of the HG generated in the design is that, despite having a diverse range of TS for various applications in tissue engineering, this susceptibility to contamination could limit its use, especially those with a higher concentration of XG or a lower number of F/T cycles.

### 2.2 Chemical structure and crystallinity

**Fig. 3:**
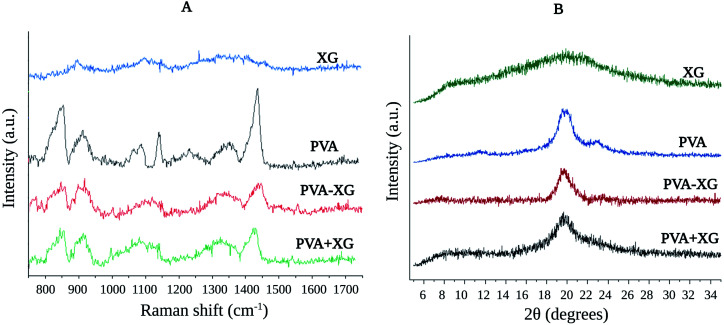
Raman spectra (A) and Diffraction patterns (B) of the optimized PVA-XG.

**Scheme 1:**
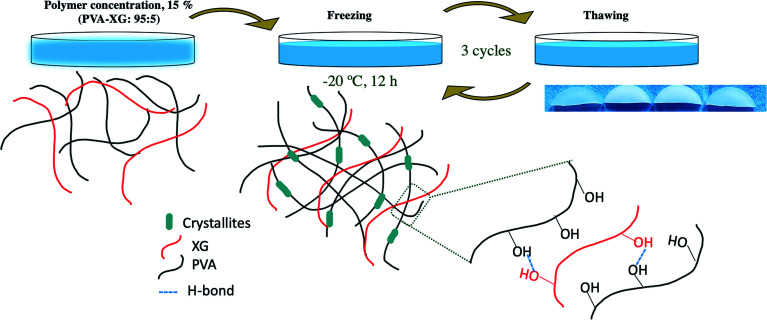
Diagram of PVA-XG hydrogel formation.

Based on Raman spectra, both PVA+XG and PVA-XG revealed characteristic signals of the PVA spectrum, associated with the amount of PVA, which is greater in the mixture than that of XG (Fig. 3A). The band at 1050–1100 cm^−1^ for pristine PVA can be attributed to O–H bond deformation vibration;^30^ this peak shows a change in both PVA-XG and PVA+XG, which may be associated with the interaction in the HG's by hydrogen bonds between PVA and XG (Scheme 1). The change is also related to a reduction in the intensity of the signal in the strong peak for PVA at 1440 cm^−1^ and has been attributed to the C–H bending and O–H bending.^31^ The two vibrational peaks for the PVA at 912 and 851 cm^−1^ are associated with the C–C stretching, and peaks at 1146 and 1045–1093 cm^−1^ are due to the C–C stretching and C–O stretching.^31^

On the other hand, XG has an amorphous structure, while PVA presents a semi-crystalline structure (Fig. 3B). Such characteristics coincide with those previously published results, where the XG exposed a characteristic peak at 2*θ* = 23.5°, which increased due to cross-linking with poly(acrylic acid).^32^ At the same time, the PVA exhibited a peak at 20°. However, the peak intensity decreased as the PVA content increased, which augmented the domain of the amorphous region.^33^ In this case, pristine PVA exposes two characteristic peaks around 2*θ* = 20.0° and 23.5°. PVA-XG presented a decrease in widening at the diffraction peak of PVA at 20.0°, not observed in PVA+XG, even that peak in PVA+XG is broader when compared with the PVA alone and with the PVA-XG, tending to approach the signal generated by the XG. However, the peak intensity at 23.5° of the PVA-XG and PVA+XG is reduced; this implies that the addition of XG into the PVA polymer matrix significantly augmented the amorphous domain of particular regions. It has been observed that XG addition and physical treatment can considerably decrease the relative crystallinity,^34^ indicating that the PVA-XG becomes much semi-crystalline. It could note that the degree of amorphous decreases when the polymer mixture is subjected to the conditions of F/T cycles (PVA-XG diffraction pattern). This change in signal is supported by the formation of crystallites (Scheme 1), which was previously mentioned concerning TS. The crystallite sizes for 2*θ* = 20.0°, based on Debey–Sherrer equation:^35^*D* = *Kλ*/*β* cos *θ* [*D* = average crystallite size, *K* = shape factor (0.89 rad), *λ*= wavelength of X-rays (1.54 Å), *β* = full width at half maximum and *θ* = Bragg angle], for PVA+XG and PVA-XG were estimated around of 1.9 and 3.4 Å, respectively.

### 2.3 Swelling behavior

HGs are polymeric materials that can swell in water and retain a significant fraction of water within their structure without dissolving.^36^ The swelling behavior of a polymer depends on several factors like the hydrophilic–hydrophobic interactions and the degree of cross-linking of the network.^37^

**Fig. 4:**
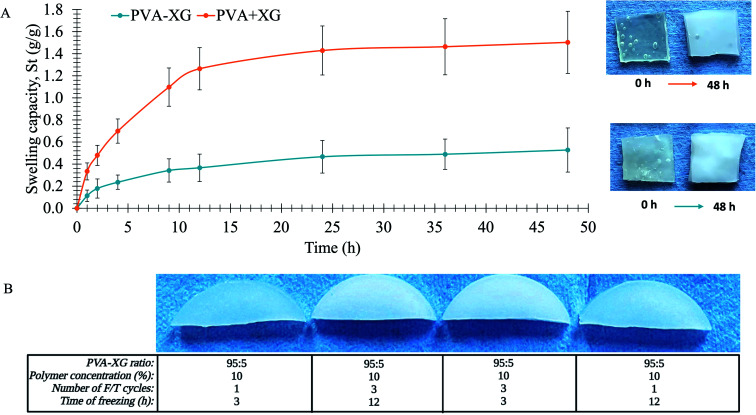
Swelling capacity of hydrogels in water at 25 °C. (A) Swelling kinetic profile of the optimized PVA-XG (mean ± CI, 95%, *n* = 3). The appearance of PVA-XG and PVA+XG before (0 h) and after the swelling study (48 h) is shown next to the profile. (B) Physical appearance of some HGs obtained during PVA-XG optimization under different experimental conditions, the TS values, from left to right, are 10.114 kPa, 51.193 kPa, 35.010 kPa, and 21.464 kPa.

The PVA-XG revealed a different swelling profile from the first hour of analysis concerning the PVA+XG, as exposed in Fig. 4A. The equilibrium swelling capacity (Se) of PVA-XG was 0.47 g g^−1^, achieved after 24 h, and a rate parameter (*r*) of 7.1 h, while the PVA+XG revealed 1.43 g g^−1^ and 6.5 h, respectively. Based on Peleg, first and pseudo-second-order models for swelling kinetics,^38,39^ the most accurate swelling process for PVA-XG and PVA+XG describes the pseudo-second-order kinetic model with determination coefficients (*R*^2^) of 0.9966 and 0.9987, respectively. This difference is associated with the crystallinity increment, related to the F/T cycles that increase the degree of cross-linking and the formation of crystallites within the system, which decreases the area permitted for diffusion across the PVA-XG network; subsequently, the capacity for PVA-XG to take up water. The incorporation of water during swelling turns the PVA-XG from a glassy phase into a rubbery phase due to the enhanced capacity of binding water and the formation of a 3D network with XG.^40^ Based on the Voigt model, the Se and *r* are the maximum water-holding capacity and the time required to reach 0.63 of the equilibrium swelling.^36^ Although PVA-XG presented a lower degree of swelling compared to PVA+XG, the strength and flexibility of the material were still adequate, and the swelling behavior, associated with the degree of cross-linking, prevented the dissolution and retained the dimensions of the network; both characteristics allow to guarantee a suitable material for spleen and bladder restoration. The increase in the cross-linking and formation of crystallites can be visually appreciated by an increase in the white appearance of the hydrogel, as exposed in different systems generated during the development of the optimized PVA-XG (Fig. 4B and Table 1). The increase in the cross-linking and formation of crystallites is due to the hydrogen bonds between PVA chains, XG chains, and PVA and XG chains. Additionally, it can be assumed that the presence of hydrophobic regions in PVA and XG causes a higher intermolecular interaction due to weak forces such as dispersion and dipole-induced dipole. Those attractive intermolecular forces are maximum due to the increase in crystallinity.

### 2.4 Thermal properties

**Fig. 5:**
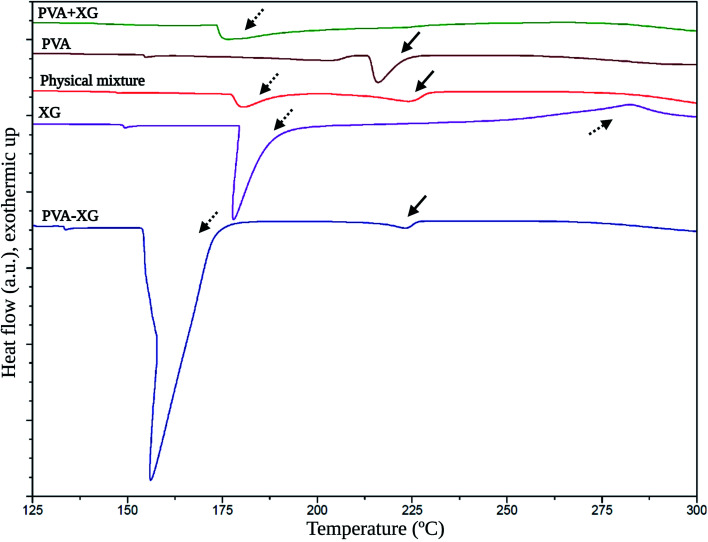
Thermal behavior of the optimized PVA-XG based on DSC. Solid arrows: thermal events related to PVA; dashed arrows: thermal events related to XG.

Fig. 5 exposes the corresponding thermograms for the raw materials, PVA+XG, PVA-XG, and the physical mixture generated with a spatula pristine PVA and XG. The XG presented an endothermic peak at 177.2 °C and an exothermic peak at 283.4 °C (dashed arrows). The endothermal lower and exothermal higher temperatures have been established as moisture loss and thermal decomposition, respectively.^41^

On the other side, PVA presented an endothermic peak at 213.5 °C (solid arrows); meanwhile, the physical mixture exhibited two thermal events at 178.3 °C and 223.4 °C, which are associated with the thermal events of the pristine XG and PVA. These events occur in lower intensity coupled with the proportions at which the polymers were mixed, and the thermal event at 178.3 °C is slightly more intense. This behavior is explained by observing that the endothermic peak of the XG at 177.2 °C is more pronounced than that presented by PVA at 213.5 °C. On the other hand, the PVA+XG exposed an event at 173.9 °C corresponding to a glass transition temperature (*T*_g_), which indicates that the mixture of XG and PVA that was not subjected to the F/T cycles generates a material with amorphous properties compared to PVA-XG, which presented an intense and broader melting temperature (*T*_m_) at 156.4 °C. Therefore, the arrangement of polymers in this system is more crystalline. Partially crystalline systems give rise to vast *T*_m_ peaks because of the size distribution of the crystallites.^42^ Furthermore, the PVA + XG did not reveal the *T*_m_ of pristine PVA at 213.5 °C, unlike the PVA-XG, which further supports the idea of material with more amorphous than crystalline properties.

The *T*_m_ exposed by the PVA-XG is mainly represented by the peak associated with the XG. However, the *T*_m_ indicates a shift towards lower values; explained by the influence of the pseudoplastic properties of XG,^43^ causing the degree of crystallinity generated by F/T cycles, and influenced by the presence of XG within the PVA-XG structure and the formation of bonds between and within polymers during the cross-linking and crystallites formation. Furthermore, XG allowed the generation of more flexible material with less mobility restriction, which coincides with studies where more elastic gels were formed at higher XG concentrations.^43^ XG chains can exist in helical conformation under low temperature, whereas they are coiled under high temperature.^44^ This transition is promoted during the heating stage in the manufacture of PVA+XG and PVA-XG. However, PVA-XG has a greater probability of recovering the helical conformation, unlike PVA+XG, due to the influence of the freezing stage at which it is subjected, which can promote its ordering and thus influence its mechanical properties.

### 2.5 Morphology

**Fig. 6:**
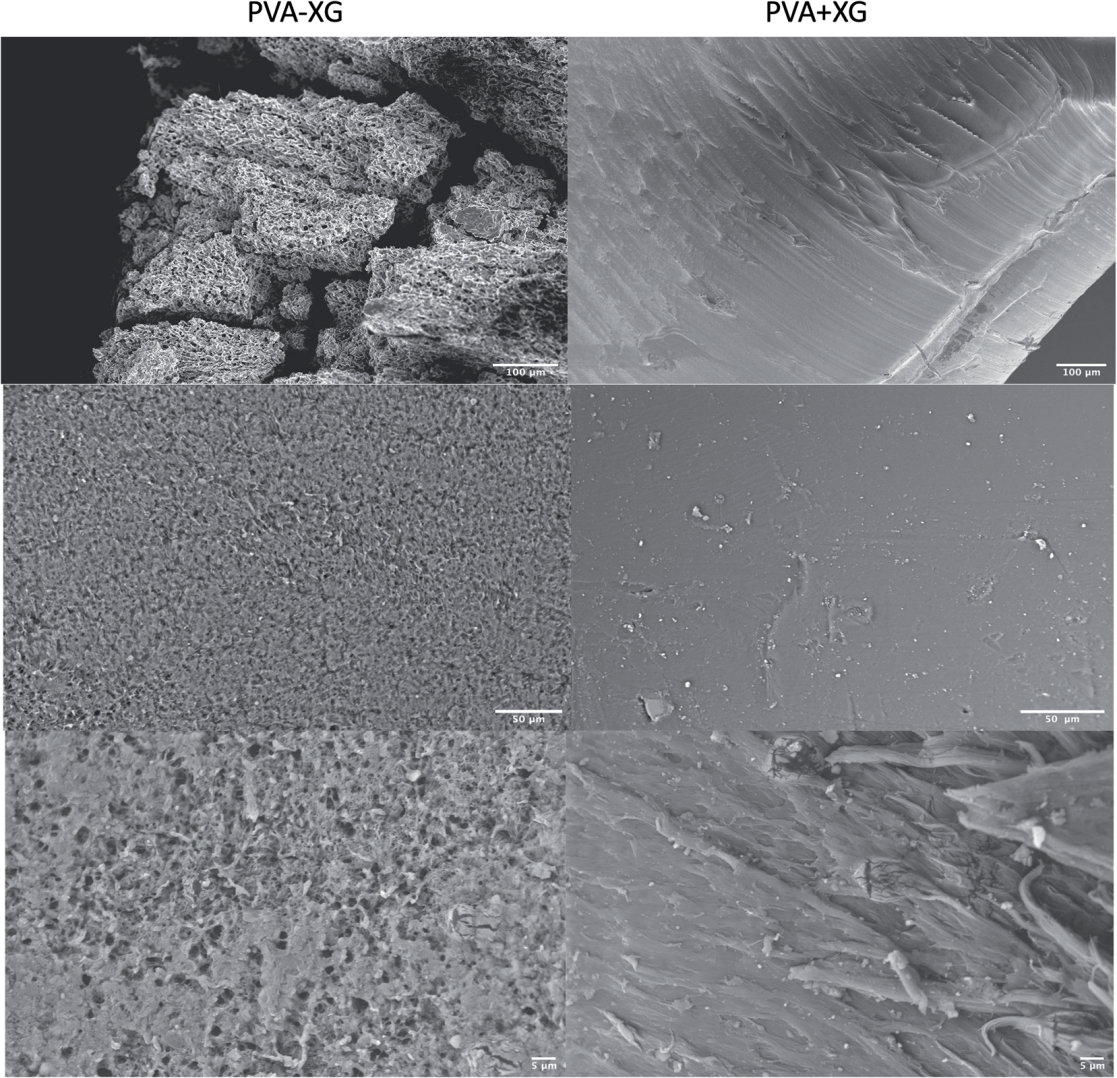
Morphology of the optimized PVA-XG (left) and PVA+XG (right) based on SEM at different magnifications.

Micrographs of the PVA-XG and PVA+XG are presented in Fig. 6. It can be observed that PVA+XG presents a smooth surface; meanwhile, PVA-XG possesses a coarser surface with a pore size of 3.02 ± 0.38 μm and a porosity of approximately 34.5% compared with PVA+XG, which is around 0.22% according to the Yuangang Zu's method.^45^ After 3 cycles of F/T, the surface of PVA-XG changes to present a rough network appearance, with a more irregular appearance, that can be explained by the formation of a physically cross-linking network in the hydrogel skeleton by PVA crystallization. This behavior has been reported before (17), showing that the F/T process increased porous morphology and promoted the formation of interconnected pores. Due to the PVA-XG HG application, these characteristics could impact the formation of new blood vessels, adequate diffusion of nutrients, suitable mass transport, and tissue formation. Furthermore, this porosity characteristic makes the hydrogel a candidate to be employed as a matrix for drug release or the incorporation of nanoparticles. It has been reported that an increase in porosity can have a beneficial effect on the diffusion of nutrients and oxygen, especially in the absence of a functional vascular system; however, the degree of porosity will also have a substantial effect on the mechanical properties, with the stiffness of the biomaterial decreasing as porosity increases.^46^ These characteristics support the porosity properties of the material, which, like the mechanical properties, are important to consider during the development of biomaterials intended for use in tissue engineering and, for PVA-XG, it is necessary to consider a deeper analysis that allows corroborating the influence of the level of porosity observed on its functional performance for the intended tissue.

Based on the observed TS values, the physically cross-linked network contributes to the chemical and mechanical properties of the PVA-XG, which is related to the results previously mentioned, such as an increase in crystallinity, a change in physical appearance, or resistance to microbial contamination.

### 2.6 Cytotoxicity assay

**Fig. 7:**
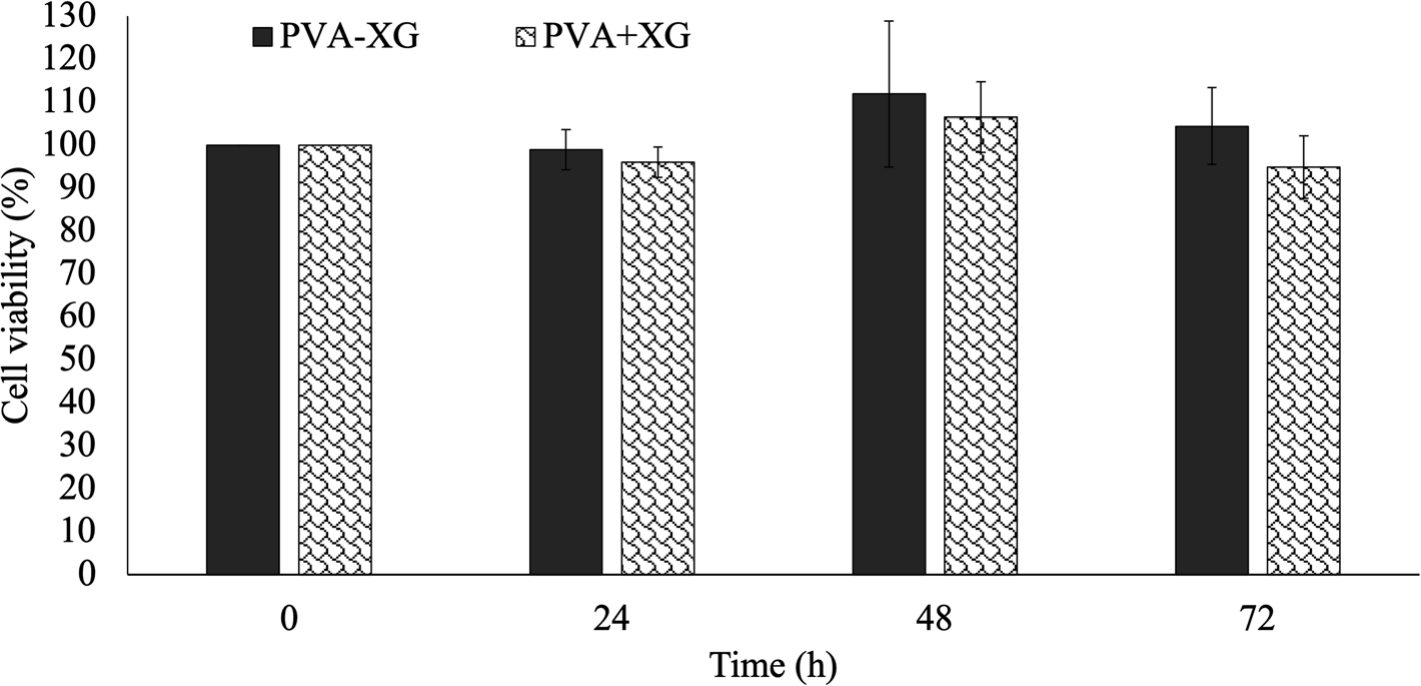
*In vitro* cell viability of dermal fibroblasts during 72 h in contact with PVA-XG and PVA+XG. Mean ± SD (*n* = 3 with 9 analysis per sample).

It has been reported that XG- and PVA-based HGs do not significantly affect cell viability. However, the combination and/or the stages involved in preparing HG could change these effects, making its evaluation essential due to the intended application.^47,48^ Based on the above, the cell viability of dermal fibroblasts in contact with PVA-XG and PVA+XG was evaluated. Fig. 7 shows the graph of the cell viability percentages obtained concerning the study time. Therefore, neither PVA-XG nor PVA+XG caused a significant decrease in the percentage of fibroblasts viability for any of the times analyzed, maintaining average percentages above 95% after 72 h. These results suggested that the PVA-XG developed, subjected to the conditions of the F/T method and in the proportion used of each polymer, is non-cytotoxic, representing an advantage for its intended application, considering that the dermal fibroblasts are distributed in the skin, which is of interest for tissue engineering.

## 3 Materials and methods

### 3.1 Materials

Polyvinyl alcohol with a hydrolysis degree of 98% and an average molecular weight range of 31 000–50 000 was purchased from Selvol™. Xanthan gum food-grade (XGF Fine Particle Size) was obtained from Jungbunzlauer®, Austria AG. The solutions were prepared using water from a Milli-Q® (MQ) filtration system (Millipore, Billerica, MA, USA).

### 3.2 Fabrication of the polyvinyl alcohol–xanthan gum hydrogel through the design of experiments

A 2^*k*^ experimental design was generated with four factors to study. Table 3 describes the factors and levels proposed for the study. Minitab® XVII software was used to establish the design matrix and respective statistical analysis. Minitab® software allows the creation of a complete factorial design with central points and, in the same way, allows to carry out an optimization of the variables.

**Table:** Factors, levels, and outcome variables for creating the matrix design

Factors (F)	Levels			Outcome variable (OV)
	Low		High	
PVA-XG ratio (% wt/wt) (F1)	85 : 15	95 : 5		Tensile strength (TS)
Number of cycles of F/T (F2)	1	3		
Time of freezing (h) (F3)	3	12		
Polymers concentration (% wt/v) in the HG (F4)	10	15		

The PVA-XG HG was prepared based on previously executed experimental strategies where the F/T method was used with other types of polymers.^17^ First, PVA powder was added into deionized water to prepare 50 ml of the solution under stirring at 85–90 °C and 700 rpm for 30 min. Then, XG powder was slowly and steadily dispersed in the system and left to swell for 24 h for complete incorporation and dispersion. After that, the mixtures were stirred at room temperature and 700 rpm for 15 min. Mixtures were poured on 5.0 cm diameter Petri dishes and left to degas for 2 h at atmospheric pressure. Finally, the samples were frozen at −20.0 ± 1.0 °C for the time established in the design. The frozen solutions were then thawed in a controlled temperature environment at 25 °C for 2 h; this F/T procedure was repeated the necessary times (Scheme 1). All the quantities and experimental conditions were based on Table 3.

### 3.3 Mechanical properties

The TS was measured as the outcome variable for each HG obtained in the design matrix. The TS test was performed using a dual-range force sensor (Vernier®). The HGs samples with a thickness of 6.57 ± 0.55 cm were sliced into rectangles (39.29 ± 1.07 cm in length and 6.64 ± 0.43 cm in width). The samples were uniaxially stretched until fractured at a controlled speed. The TS was calculated by TS = Fo/w*t, where Fo is the loading force, and *w* and *t* are the original width and thickness of the specimen, respectively. The Fo was recorded as the force necessary to break the HG on the central zone. The TS determination was performed directly on the HGs obtained after F/T cycles.

### 3.4 Characterization of the optimized hydrogel

Once the HG with the best mechanical properties was found, it was characterized by the following tests using lyophilized HG (PVA-XG) and compared with an HG coded as PVA+XG. PVA+XG was prepared with the same ratio and polymer concentration of the optimized PVA-XG (Table 2) and following the methodology mentioned in the second part of section 3.2 but without subjecting it to any cycle of F/T. The PVA+XG was also lyophilized.

### 3.5 Raman spectroscopy

Raman spectra were acquired by Thermo Scientific® TruScan analyzer (RM, Witec, Germany) to evaluate the possible chemical changes associated with the F/T cycles. Samples of raw materials, PVA+XG and PVA-XG, were excited with an LED laser source of 250 mW, at 785 nm and with a line width of 2.0 cm^−1^. The spectrum range analyzed was 700 to 1800 cm^−1^.

### 3.6 X-ray diffraction (XRD)

In order to confirm a change in the crystallinity of PVA-XG, an XRD-Bruker® D8-Advance (Ettlingen, Germany) X-ray diffractometer was used to analyze the XRD pattern of raw materials, PVA+XG and PVA-XG at room temperature. The sample analysis was performed with the Cu-Kα radiation at the wavelength of 1.54 Å working at 40 kV in the scanning angle range of 2*θ* = 5°–45° with a Lynxeye silicon strip detector.

### 3.7 Swelling test

Crosslinking of polymer chains can modify the response to the HGs; therefore, a swelling profile was measured. For the swelling test, 0.5 g of sample was put into a meshed diffusor thee capsule and was introduced into the water at 25 °C with a constant stirring at 50 rpm and taking different samples in the interval of 1–48 h. After each sample, the sample weight of the previously blotted with a filter paper was determined, and finally, the sample was returned to the water. This procedure was repeated as many times as necessary until the increase in swelling capacity became constant. The test was carried out in triplicate. Finally, both profiles (PVA-XG and PVA+XG) were analyzed based on Peleg, first and pseudo-second-order models for swelling kinetics^38,39^ according to the following models:Peleg: *t*/[*Qt* – *Qe*] = *k*_1_ + *k*_2_*t*First order: ln (*Qe* − *Q*_*t*_) = ln(*Qe*) − *k*_1_*t*Pseudo-second order: *t*/*Qt* = [1/*k*_2_*Q*_*e*_] + *t*/*Q*_*e*_where *Q*_e_, *Q*_t_, *k*_1_, and *k*_2_ are defined as swelling content at equilibrium, swelling content at the time (*t*), and swelling kinetic constants, respectively.

### 3.8 Differential scanning calorimetry (DSC)

HG samples of about 10 mg were tested using a differential scanning calorimeter (DSC, TA Instrument™, Q20) to determine the presence of thermal events associated with amorphous and crystalline materials and contrast with those obtained in the XRD studies. The samples were collocated in aluminum hermetic crucibles. Measurements were carried out under a 50.0 ml min^−1^ nitrogen flow in the temperature range from 125 °C to 300 °C at a heating rate of 10 °C min^−1^. Before heating, the samples were kept in equilibrium with an isotherm at 20 °C for 2 min.

### 3.9 Scanning electron microscopy (SEM)

The morphology and microstructure of the HGs were characterized by scanning electron microscopy, SEM (EVO 10, Zeiss®, Oberkochen, Germany), and observed by using an accelerating voltage of 15 kV after sputter coating with gold under vacuum. Specimens were mounted perpendicularly to their surface on aluminum stubs and fixed using carbon tape.

### 3.10 Determination of porosity of PVA-XG HGs

The porosity of the PVA+XG and PVA-XG was measured based on Yuangang Zu's method.^45^ First, the weight of an empty 10 ml beaker (*W*_1_) was measured, then 1.0 ± 0.3 g of the HG was placed into the beaker, and cyclohexane was slowly poured in. Next, the weight (*W*_2_) of the beaker was then measured. After that, the HG was removed and weighed (*W*_3_). The HG pores were full of cyclohexane, and the volume of cyclohexane in an HG pore was taken as the pore volume of the HG. The porosity of the HG (*P*) was calculated using the following equations:
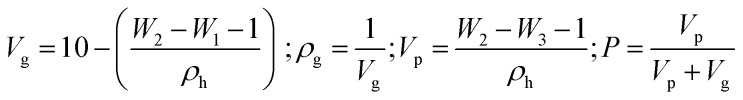
where *ρ*_g_ is the density of the hydrogel (g cm^−3^), *ρ*_h_ is the density of the cyclohexane (g cm^−3^), and *V*_g_ is the volume of the hydrogel (cm^3^), and *V*_p_ is the volume of cyclohexane in the pore (cm^3^).

### 3.11 Cytotoxicity

The development of non-cytotoxic materials is of interest in the tissue engineering field; therefore, to evaluate the cytotoxicity of HG, a cell viability test was performed on dermal fibroblasts derived from a healthy adult. A Cell Proliferation Kit I MTT kit (Cat. 11465 007 001, Roche Diagnostics® GmbH) was used. Cells were seeded at 70% confluence in 96-well plates incubated at 37 °C, 5.0% CO_2_, in a humid environment for 24 h. Then the medium was removed, and two washes were performed with 100 μl of PBS. Subsequently, 100 μl of MEM (Cat. 41500-018, Gibco®) supplemented with 10% of FBS and 50 U ml^−1^–50 μg ml^−1^ Pen/Strep (Cat. S1820 Biowest®, Cat. 15070063 Gibco®) together with 2 mg of PVA-XG or PVA+XG, were incubated for 24, 48 and 72 h. Once the incubation time was over, the HGs were removed, and 10 μl of MTT (final concentration 0.5 mg ml^−1^), incubated for 4 h, were added. Afterward, formazan crystals were dissolved with 100 μl of solubilization buffer in each well. The absorbances were determined at 595 nm OD with an iMark plate reader (Bio-Rad Laboratories®, Inc).

### 3.12 Statistics

The Minitab® XVII computer program was used to perform statistical analysis. The influence of different factors on TS and the optimization of PVA-XG was performed by analysis of variance (ANOVA), while the difference between means was assessed using the *t*-student test, with a significance level set at *p* ≤ 0.05.

## 4 Conclusions

This study developed and characterized, through a 2^*k*^ factorial design strategy that allowed the evaluation of several factors involved in the F/T technique, hydrogels based on polyvinyl alcohol and xanthan gum with tensile strengths ranging from 9 to 87 kPa, which are appropriate for developing scaffolds of organs such as cardiac and skeletal muscle, dermis, thyroid, bladder, and spleen. The optimized PVA-XG at 95 : 5 ratio showed adequate mechanical, chemical, and biological properties for potential tissue engineering applications. These properties were obtained with a polymer mixture concentration of 15% and 12 h of freezing with three cycles of freeze/thaw. This allowed obtaining a material with higher crystalline properties, flexible and resistant. However, it is important to mention that, regardless of the existence of a range of possible TS based on the generated design, the additional characteristics, such as swelling capacity or cytotoxicity, that were presented for the PVA-XG hydrogel would have to be evaluated as an alternative of the design strategy to guarantee that the modifications in the different factors studied allow obtaining an optimal HG for the predefined potential use.

The new hydrogel exhibited a pore size of 3.02 ± 0.38 μm and a porosity of approximately 34.5%. Likewise, it was observed that the number of freeze/thaw cycles, the freezing time, and the proportion of xanthan gum in the hydrogel influence the microbiological contamination, which also becomes an important outcome variable to be measured during the development of this type of system and especially with HGs where the XG concentrations are high and/or a lower number of F/T cycles. Finally, the hydrogel developed did not show a cytotoxic effect when placed on a fibroblast culture, enhancing its use as an alternative in biomaterials used in tissue engineering. However, it is necessary to carry out *in vivo* studies to corroborate the safety and efficacy of the optimized HG, as well as more in-depth characterization studies that allow corroborating and complementing the mechanical behavior such as elastic modulus and the physical and chemical characteristics of the biomaterial.

## Author contributions

Conceptualization, S. A. B.-C. and G. L.-G.; methodology, S. A. B.-C., S. A.-A., Y. S. T.-G., J. J. M., M. L. D. P.-A., and G. L.-G.; software, S. A. B.-C. and S. A.-A.; validation, S. A. B.-C., and S. A.-A.; formal analysis, S. A. B.-C., M. L. D. P.-A., and G. L.-G.; investigation, S. A. B.-C.; resources, S. A. B.-C., S. A.-A., and Y. S. T.-G.; data curation, S. A. B.-C.; writing—original draft preparation, S. A. B.-C.; writing—review and editing, S. A. B.-C., M. L. D. P.-A., and G. L.-G.; visualization, S. A. B.-C. and G. L.-G.; supervision, M. L. D. P.-A. and G. L.-G.; project administration, G. L.-G.; funding acquisition, G. L.-G.

## Conflicts of interest

There are no conflicts to declare.

## Supplementary Material
